# Changes to the law on consent in South Africa: implications for school-based adolescent sexual and reproductive health research

**DOI:** 10.1186/1472-698X-12-3

**Published:** 2012-04-10

**Authors:** Melanie Zuch, Amanda J Mason-Jones, Catherine Mathews, Lesley Henley

**Affiliations:** 1 Brown University, Medical Research Council, PO Box 19070, Cape Town, Tygerberg 7505, South Africa; 2 Adolescent Health Research Unit, University of Cape Town, Specialist Scientist, Health Systems Research Unit, Medical Research Council, PO Box 19070, Cape Town, Tygerberg 7505, South Africa; 3 School of Public Health and Family Medicine, University of Cape Town, Health Systems Research Unit, Medical Research Council, PO Box 19070, Cape Town, Tygerberg 7505, South Africa; 4 School of Child and Adolescent Health, University of Cape Town, Cape Town, Rondebosch 7700, South Africa

## Abstract

**Background:**

The National Health Act, No 61, 2003 in South Africa is the first effort made by the government to protect health-related research participants under law. Implemented on March 1, 2012, the law mandates active consent from a parent or legal guardian for all research conducted with research participants under the age of 18 years. This paper focuses on the Act's implications for school-based adolescent sexual and reproductive health research.

**Discussion:**

Although well intentioned, the added legal protections in the National Health Act may have the unintended consequence of reducing participation rates in school-based adolescent sexual and reproductive health research, thereby excluding the most at-risk students. The Act may also compromise adolescents' right to dignity and privacy, especially considering the personal nature of research on sex and sexuality. Devolved, discretionary decision-making, which empowers local human research ethics committees to permit a wider range of protective measures, including passive consent, independent adolescent consent or community consultation ought to be considered. The continued and direct involvement of young people in their sexual and reproductive health and well-being is an important principle to uphold.

**Summary:**

This paper calls for a re-examination of section 71's ethical guidelines relating to informed consent in the National Health Act, No 61, 2003 in South Africa in order to better serve the interests of South African adolescents in sexual and reproductive health research.

## Background

Protecting the welfare of research participants, particularly vulnerable groups such as adolescents, is of primary importance to any researcher to ensure that research-related harm and exploitation are avoided. However, a recent audit commissioned by the Vulnerable Subjects Working Group of the National Health Research Ethics Council (NHREC) found that prior to the implementation of the National Health Act No 61, there was limited specific legal protection for vulnerable research participants in South Africa [1]. Before the implementation of the Act, the legal framework did not specifically address health-related research with adolescents or other vulnerable groups at all, leading research ethics committees to rely on guidance from general principles such as laws relating to the medical treatment of ‗minors'.

It would seem then that the National Health Act No 61 of 2003 [2] and its research-specific provisions (s71), enacted on March 1, 2012, would be welcomed wholeheartedly by researchers, but a closer look at the provisions contained therein belies the potentially far-reaching consequences for conducting school-based adolescent health research in South Africa. The purpose of this paper is to explore these proposed new provisions in terms of the effects on adolescent health research and to suggest that these provisions do not take into account the emerging autonomy of the adolescent sufficiently nor the current South African social context and make suggestions that will optimize the balance between adolescents' access to research and their protection as research subjects.

## The South African regulatory framework

Prior to March 1, 2012, the framework governing research in South Africa permitted adolescents to consent independently to take part in research. In -Ethics in Health Research: Principles, Structures and Processes," the South African Department of Health states that adolescents, defined as persons who have reached puberty, may consent unassisted to research so long as: (i) the research poses --no more than minimal risk" to the adolescent; (ii) --the parents or legal guardians or community at large are unlikely to object" to the adolescent's participation; (iii) the protocol justifies --why adolescents should be included as participants"; and (iv) the protocol justifies --why the adolescent participants should consent unassisted" [3]. In contrast, the provision of Section 71 of the National Health Act No 61, 2003, does not make the same distinctions between children and adolescents with regards to independent consent. The Act is the first effort made by the South African government to protect research participants under law [4]. Section 71 of the Act mandates *active written consent *from a parent or legal guardian for all research conducted with subjects under the age of 18. The Act says: --where research or experimentation is to be conducted on a minor for a therapeutic purpose, the research or experimentation may only be conducted--(a) if it is in the best interests of the minor; (b) in such manner and on such conditions as may be prescribed; (c) with the consent of the parent or guardian of the child; and (d) if the minor is capable of understanding, with the consent of the minor." For non-therapeutic research and experimentation, it may only be conducted, --(i) in such manner and on such conditions as may be prescribed; (ii) with the consent of the Minister (of Health); (iii) with the consent of the parent or guardian of the minor; and (iv) if the minor is capable of understanding, the consent of the minor" [2]. No other caregiver or custodian will be able to give consent for a child's participation in research [5].

Thus, active consent from a parent or legal guardian is the only way that subjects under the age of 18 can participate in research in South Africa [2].

Of particular concern is wide definition of -health research" in the Health Act. According to the definition, -health research" includes (among others) -any research which contributes to knowledge of the biological, clinical, psychological or social processes in human beings" [2]. Arguably, social science research will then need to meet similar consent requirements as those laid down for medical research.

While the intentions of the National Health Act are to provide legal protection to research participants, the Act leaves no room for independent adolescent consent for studies exploring their sexual and reproductive health. Indeed, the mandate of active parental consent under the Act may greatly reduce participation rates and introduce recruitment bias to school-based adolescent sexual and reproductive health research projects.

## Consent for adolescent sexual and reproductive health research

The issue of participant informed consent remains central to any research project in the medical and social sciences. The fundamental principle stated in the Declaration of Helsinki is a person's right to make an autonomous informed decision about his or her participation in research [6] whilst the first point in the Nuremberg Code deems informed consent to be -absolutely essential" [7]. However, ambiguity surrounds questions of who can consent for young adolescents, what type of consent is most appropriate, and at what point that young person can consent for him or herself [8]. In the process of protecting adolescents from exploitative or undesired research, there exists a tension between the protection of research subjects, and excessive regulations that may disrespect the autonomy of the individual, or result in such research being unfeasible [9].

This debate is especially acute in adolescent sexual and reproductive health research [10], which is often conducted to develop and evaluate effective interventions to reduce sexual risk behavior. Indeed, the scientific and ethical review group at the World Health Organization states in their guidelines for adolescent reproductive health research that omitting this type of research can -perpetuate inadequate understanding of the particular reproductive health needs of adolescents and result in failure to deliver adequate services to this group" [11]. This type of research typically takes the form of surveys or classroom-based interventions and is conducted with high school students who are in general 12 years and older. Such studies have been deemed to be no more than minimal risk; in other words, the risks associated with daily life or routine medical and psychological examinations. Indeed, recent research shows that most adolescents do not report increased distress when completing surveys which address sensitive topics, which include questions about suicidal behavior, illicit drug use and physical and sexual abuse [12].

## Issues in parental consent

There are generally two primary forms of parental consent used in social science and health services research with adolescents. Active consent necessitates a ‗yes' response from the parent, in order that a young person may participate in research. Passive consent assumes the parent's affirmative response, unless the parent indicates otherwise [13,14]. It is assumed that active consent comes with greater confidence that a parent has actually read and understood the details of the research project, whereas with passive consent, a parent's lack of response could result from their failure to receive, read or understand the letter rather than their willingness to allow the child to participate [13]. Active consent is also assumed to bring a sense of parental involvement in the research project, which can be important to interventions that aim to reduce rates of adolescent sexual risk behavior and/or to change social norms. However, active parental consent--often denoted by a signature on a form--is not always a true indication of an adult's understanding of or involvement in the research in question [15,16].

A study in South Africa demonstrated that despite an apparent active parental consent response rate of 94%, only 65% of parents had received the information letter and consent form during a follow-up interview. Many parents also reported being unaware of the letter's contents, despite having signed the consent form. Follow up interviews were conducted with 18 parents who denied consent for their child to participate; 15 of these parents actually did want their children to participate. Of the 12 parents who were interviewed after not responding to the letter sent home, all 12 had not received the consent letter, and said they also wanted their child to participate in the research[16]. It is not surprising therefore, that active parental consent can generate low response rates of only 30%-60% [14]. But it may not necessarily reflect parents actively refusing to allow their children to participate in the research. Oftentimes non-response is a result of a parent's not receiving the letter, or a lack of understanding [17].

Failure to understand an informed consent letter may be a particular problem in South Africa, where high illiteracy rates are prevalent [18,19]. Parents may not feel comfortable signing or finger printing a document that they cannot fully comprehend or their signature may not be a valid sign of their comprehension [16]. Beyond literacy rates, cultural and language differences can impede informed consent, as can the effect of a power imbalance between the researcher and the subject, and subject's parent [19].

Requiring active parental consent procedures may also hinder research by adding a prohibitive cost to research [20,21] and by introducing a potentially significant sample bias into the data [17,22]. A study of parental consent procedures in the United States demonstrated that students who gained consent from their parents were more likely to be female, White, from intact homes, less likely to smoke cigarettes, and have parents who were more likely to be educated [14]. In this way, students at greater risk for poor health are likely to be disproportionately excluded by active consent procedures [13,14]. This sample bias not only poses a problem for research integrity; it also may prevent research from reaching the students who stand most to benefit from it.

The new Act specifically mandates consent from a *legal *guardian, despite the fact that many children in South Africa do not live with a parent or a legal guardian, but rather with another caretaker or custodian. According to a report by the South African Human Rights Commission and UNICEF, only 32% of South African children live with both of their biological parents and 19% have lost one or both parents [23]. Additionally, UNICEF estimates that there are 3.7 million orphans in South Africa [24], and according to a study by the South African Institute of Race Relations, 98,000 children were living in child-headed households as of 2008 [25]. Whilst the exact number of children without a legal guardian is unknown, these statistics suggest that the number is high, as children who have lost their parents may be living under the auspices of another adult who has not legally adopted them.

We suggest that laws governing research in South Africa must consider its unique social circumstances. The National Health Act will prevent children without a legal guardian from accessing research that could be of great benefit to them in terms of reducing rates of sexual risk behavior, pregnancy and STI/HIV transmission. This is especially problematic given that orphans and vulnerable children, precisely those without legal guardians, are at an elevated risk of contracting HIV and other STDs, and becoming pregnant [26].

## Independent adolescent consent

Early empirical evidence has shown that young adolescents may be as competent as adults in their ability to provide informed consent in terms of -stringent legal standards of competency" [27]. While an adolescent's ability to make competent decisions does vary from person to person, this evidence compels a deeper look into the option of independent consent for school-based research with adolescents aged from 12 years, particularly if procedures are in place for research staff to gauge the subjects' levels of understanding. Such an assessment may include verbal or written tests to gauge the participant's level of understanding of the consent form, understanding of confidentiality procedures, and knowledge of their rights as research subjects as well as their rights to withdraw [28].

In some cases, children as young as 8 years old have been deemed competent to make autonomous decisions in medico-legal matters [29] and from 5 years in research [30]. An adolescent's ability to provide informed consent can depend on individual emotional maturity level, their perceived or actual ability to make decisions in their day-to-day lives (which can be dependent on cultural and/or familial norms), reasoning skills, memory and language [29]. Interestingly, the Children's Act of South Africa sets the minimum age of independent consent for medical treatment and surgical operations at 12 years [31]. If consistent standards were applied to adolescent participation in research, active parental consent would not be mandatory as long as the subject were deemed able to consent for him or herself. Passive parental consent or community-based collaborative consent may mitigate any potentially negative consequences of active procedures, and potentially grant adolescents more autonomy during research participation. This is particularly important in research into adolescent sexual and reproductive health, which oftentimes includes sensitive and personal topics that an adolescent may not want to share with his or her parents.

Adolescents are in the process of becoming fully autonomous individuals [32] and although an adolescent's degree of autonomy and cognitive ability to consent will differ from individual to individual, it is generally accepted that 12-year-olds are old enough to make autonomous and informed decisions about their participation in a school-based adolescent sexual research project which poses minimal risk to them [27].

## Discussion

Requiring active parental consent may not be appropriate for all study settings and populations, particularly where cultural norms differ from Western ethical standards [19]. Concerns with the new guidelines are not limited to the tangible ways in which they may hinder the implementation of research. In prohibiting students aged 12-18 years from providing independent consent under any circumstances, the enactment of Section 71 of the National Health Act No 61, 2003 may be compromising a child's right to dignity and privacy [4,33]. Confidentiality is a central tenant of research ethics, especially in research surrounding sensitive topics such as sexuality. In South Africa in particular, discussions surrounding sexuality are often shrouded in stigma [34] and parent-child communication with regards to sex and sexuality is often limited [32]. An adolescent therefore may not feel comfortable confronting a parent or guardian about participation in a sexual and reproductive health research study or may face disapproval if he or she chooses to do so [32]. If a parent interprets an adolescent's desire to participate in a sexual and reproductive health study as proof that their child is sexually active, the child could lose access or knowledge of sexual and reproductive health services due to stricter parental supervision [10].

We believe that adolescents from the age 12 onwards should be allowed to participate in school-based sexual and reproductive health research, which recognizes and seeks to understand their unique set of needs in order to develop programs to keep them healthy even if their parents would choose to deny them access on moralistic grounds. It is important to note that according to current South African research ethics guidelines any human research must receive prior approval by a relevant human research ethics committee in order to ensure an adequate balance of risks and benefits regardless of whether their parents consented to their participation. Moreover, in the case of school-based research, the approval of the Department of Education and the school principal is also needed before implementation.

Social realities in South Africa must be reflected in laws relating to research among adolescents about their sexual and reproductive health. The Department of Health's prior ethical guidelines [3] appear sufficient whilst the new changes appear to be inconsistent with other guidance and are retrogressive. It is acknowledged that parental involvement is critical to an adolescent's wellbeing, and programs to improve adolescent sexual and reproductive health should actively involve parents in the planning stages and encourage improved parent-child communication.

Several researchers have rightly asserted that age is a very limited measure of an adolescent's cognitive ability to fully understand and consent to research [29,30] and that adolescents are developmentally more inclined to underestimate the effects of taking a risk, and thus cannot necessarily be deemed capable of making informed decisions about participation in research [34]. In this regard, other methods of obtaining collaborative adult consent must be considered, including obtaining active parental consent at the beginning of the school year during registration to better ensure comprehension and response [16], as well as methods that embody a process of community engagement whose principles are found at the heart of rural communities in South Africa [35,36]. One possibility is to consult community forums to gain consent for research projects, instead of gaining consent from parents only [35,37]. Such group consent procedures can facilitate the process of decision-making, ensuring that the study is understood and supported by the community as well as by individual participants [38]. Significantly, this principle of community consultation already underpinned the Department of Health's prior ethical guidelines. Other alternatives include the inclusion of adolescents on research ethics committees [39], and/or the establishment of Youth Advisory Committees, in which adolescents themselves can inform the development, content and implementation of research [10,40].

## Summary

Adolescent sexual and reproductive health research in South Africa is of great importance, as teenage pregnancy, sexually transmitted disease and HIV-rates remain high in this population [41]. Maintaining high standards in our ethical approach is critical to protect vulnerable research subjects from potential harm. However, to preserve the right of young people to self-determination, we call for a re-examination of the National Health Act guidelines for research. The proposed enactment may actually undermine the rights of young people in South Africa.

## Competing interests

MZ is a law student, AMJ and CM are involved in adolescent health research, AMJ was previously a member of a research ethics committee and LH is a current member of a research ethics committee.

## Authors' contributions

AMJ conceived of the idea, and MBZ researched and drafted the first version of the paper. All authors revised for content and style, and all have read and approved the final manuscript.

## Pre-publication history

The pre-publication history for this paper can be accessed here:

http://www.biomedcentral.com/1472-698X/12/3/prepub
